# Deletion of TSPO Causes Dysregulation of Cholesterol Metabolism in Mouse Retina

**DOI:** 10.3390/cells10113066

**Published:** 2021-11-07

**Authors:** Fahad Farhan, Mohammad Almarhoun, Aileen Wong, Amy S. Findlay, Chris Bartholomew, Mark T. S. Williams, Toby W. Hurd, Xinhua Shu

**Affiliations:** 1 Department of Biological and Biomedical Sciences, Glasgow Caledonian University, Glasgow G4 0BA, UK; fahad.farhan@gcu.ac.uk (F.F.); MALMAR200@caledonian.ac.uk (M.A.); Aileen.Wong@gcu.ac.uk (A.W.); C.Bartholomew@gcu.ac.uk (C.B.); Mark.Williams@gcu.ac.uk (M.T.S.W.); 2 MRC Human Genetics Unit, Institute of Genetics and Cancer, University of Edinburgh, Edinburgh EH4 2XU, UK; Amy.Findlay@igmm.ed.ac.uk (A.S.F.); toby.hurd@ed.ac.uk (T.W.H.); 3 School of Basic Medical Sciences, Shaoyang University, Shaoyang 422000, China; 4 Department of Vision Science, Glasgow Caledonian University, Glasgow G4 0BA, UK

**Keywords:** TSPO, retina, cholesterol, inflammation, age-related macular degeneration

## Abstract

Cholesterol dysregulation has been implicated in age-related macular degeneration (AMD), the most common cause of visual impairment in the elderly. The 18 KDa translocator protein (TSPO) is a mitochondrial outer membrane protein responsible for transporting cholesterol from the mitochondrial outer membrane to the inner membrane. TSPO is highly expressed in retinal pigment epithelial (RPE) cells, and TSPO ligands have shown therapeutic potential for the treatment of AMD. Here, we characterized retinal pathology of *Tspo* knockout (KO) mice using histological, immunohistochemical, biochemical and molecular biological approaches. We found that *Tspo* KO mice had normal retinal morphology (by light microscopy) but showed elevated levels of cholesterol, triglycerides and phospholipids with perturbed cholesterol efflux in the RPE cells of *Tspo* KO mice. Expression of cholesterol-associated genes (*Nr1h3*, *Abca1*, *Abcg1*, *Cyp27a1* and *Cyp46a1*) was significantly downregulated, and production of pro-inflammatory cytokines was markedly increased in *Tspo* KO retinas. Furthermore, microglial activation was also observed in *Tspo* KO mouse retinas. These findings provide new insights into the function of TSPO in the retina and may aid in the design of new therapeutic strategies for the treatment of AMD.

## 1. Introduction

Age-related macular degeneration (AMD) is the most frequent condition leading to legally defined blindness among senior citizens. In early stages of the disease, AMD patients may encounter no or limited vision loss. Unfortunately, as the disease progresses, they may experience the total loss of central vision, finally resulting in complete blindness. In practice, patients may discern no significant disturbance to peripheral vision, but the condition can have an adverse effect on their ability to read or drive [1]. The disease is seldom detected in people under 50 years of age, but the prevalence rises with aging, with 60% of people over the age of 90 affected by AMD [2,3]. Late AMD is divided into two subgroups: dry (geographic atrophy, accounting for 90% of AMD) and wet (neovascular) AMD. Anti-vascular endothelial growth factor (VEGF) drugs demonstrate protective effect in treating wet AMD. However, there is no effective treatment for dry AMD patients [4].

The generation of superfluous energy associated with abnormal lipid metabolism can cause dyslipidaemia, which is a crucial precursor to cardiovascular disease, obesity and cholestasis [5]; it also constitutes a significant risk factor for the advancement of AMD [6]. Cholesterol, the principal sterol, accumulates in deposits known as drusen, in the retinas of AMD patients [7,8]. In addition, a diet rich in cholesterol and saturated fat constitutes a high risk for development of AMD [9]. Certain genetic variants of cholesterol homeostasis genes are often associated with AMD [10]. Preclinical examination demonstrates that lipid dysregulation can boost the progress of AMD lesions in animal models which replicate certain phenotypes of AMD [11,12].

Translocator protein (TSPO) was initially identified in 1977 as a peripheral benzodiazepine receptor [13]. TSPO is a 169-amino acid protein localized to the outer mitochondrial membrane and is encoded by a nuclear gene containing four exons [14]. TSPO contains five α-helical transmembrane domains and includes a cholesterol-recognition amino acid consensus (CRAC) sequence at its C-terminus which bends toward the lipid membrane [15]. TSPO is thought to be involved in mitochondrial transition pore regulation, steroid synthesis, Ca^2+^ homeostasis, the generation of reactive oxygen species and the production of energy from nutrients [14,16]. Following identification of TSPO as a peripheral binding site for benzodiazepines, TSPO ligands were investigated as possible treatments for psychiatric disorders. Indeed, these ligands brought about positive results for anxiety maladies in animals and human subjects [14,17]. It has been postulated that TSPO ligands increase levels of (neuro)steroids, such as allopregnanolone, and as a consequence, moderate the activity of GABA_A_ [17]. TSPO is highly expressed in steroidogenic tissues including the gonads, adrenal glands and the cerebrum [16]. Accordingly, the major function of TSPO is mitochondrial cholesterol trafficking, transporting cholesterol from the mitochondrial outer membrane to the inner membrane, where cholesterol is metabolized into steroids in steroid-generating cells or oxysterols in nonsteroidogenic cells [18].

Our previous studies demonstrated that TSPO is expressed at high levels in the retinal pigment epithelial (RPE) and choroidal endothelial cells and regulates cholesterol efflux [18,19]. Aging mice exhibit decreased expression of *Tspo* and reduced cholesterol efflux in the RPE cells. TSPO deletion leads to increased uptake and accumulation of oxidized low-density lipoprotein (LDL), and higher production of reactive oxygen species and proinflammatory cytokines [18]. Additionally, we found that TSPO ligands promoted cholesterol efflux in RPE and choroidal endothelial cells and decreased lipogenesis [18,19]. A TSPO ligand, etifoxine, also decreased serum and RPE cholesterol in mice fed with a high-fat diet and lowered inflammatory cytokines in serum and the RPE [20,21]. In this work, we have characterized the *Tspo* knockout (KO) mice, specifically examining retinal histology and cholesterol homeostasis during aging.

## 2. Materials and Methods

### 2.1. Animals

All animal work was carried out in compliance with the Animal Ethics and Welfare Committee, Glasgow Caledonian University, and the UK Home Office under a Project License PPL 60/4347. The *Tspo* floxed (wildtype, WT) and *Tspo* knockout (KO) mice were gifted from Dr. Vimal Selvaraj (Cornell University) [22,23]. All animals were housed under a standardized light–dark cycle and all efforts were applied to use a minimum number of animals and to ensure minimum suffering.

### 2.2. Genotyping

DNA was extracted from mouse ear notch and dissolved in sterile dH_2_O. Polymerase chain reaction (PCR) was performed by using the DreamTaq PCR Reddy Master mix (Thermo Fisher Scientific, Paisley, UK), following the manufacturer’s protocol. Each PCR reaction contained 25 µL DreamTaq PCR Reddy Master mix, 1 µL, 100 µM of forward (5′TCACCAAGGGTGTGAATGAA3′) and reverse (5′AACCTACCTGGTGGCTTCCT3′) primers, 1 µL mouse DNA and 22 µL of nuclease-free water. The thermo-cycle program for PCR is 94 °C for 3 min, 40 cycles of 94 °C for 15 s, 60 °C for 15 s and 72 °C for 1 min 10 s, and 72 °C for 7 min. The PCR products were separated in 1% agarose gel.

### 2.3. Western Blotting

Brain, retinas and RPE/choroid/sclera were dissected from wildtype and *Tspo* KO mice, then homogenized in cold T-PER reagent (Thermo Fisher Scientific, Paisley, UK). The tissue lysates were centrifuged at 10,000× *g* for 10 min. The supernatants were collected and the concentration was measured. Then, 50 µg proteins from each sample were separated by sodium dodecyl sulphate–polyacrylamide gel electrophoresis (SDS–PAGE) and transferred into nitrocellulose membrane. The membrane was initially blocked with 5% non-fat dry milk powder in PBS, then incubated with primary antibodies and corresponding secondary antibodies respectively. Targeted protein signals were detected using the LI-COR Odyssey FC Imaging System.

### 2.4. Haematoxylin and Eosin Staining (H&E)

Mouse eyes were fixed with 4% paraformaldehyde (PFA) in phosphate-buffered saline (PBS) (Thermo Fisher Scientific, Paisley, UK), then washed by PBS twice, followed by dehydration through 5%, 15% and 30% sucrose. The eye samples were embedded in Optimal Cutting Temperature (OCT) compound (VWR, Lutterworth, UK) and cut into 8 μm–10 μm thickness. The cryosections were further fixed by 100% cold methanol for 30 min at −20 °C. The slides were stained with hematoxylin (Sigma, Dorset, UK) for 8 min, then washed for 20 min with running tap water and rehydrated through 50% ethanol for 2 min and 70% ethanol for 2 min, finally counterstained by Eosin (Sigma, Dorset, UK) for 1 min. The slides were further dehydrated by passing through 90% ethanol for 1 min and 100% ethanol for 5 min. Slides were photographed under light microscope (Olympus, Essex, UK). For measuring the thickness of the retinal outer nuclear layer (ONL), two retinal sections from each eye were chosen and images were captured from the superior and inferior sides of the optic nerve head; the thickness of ONL was measured at intervals of 200 µm. Four eyes from four individual animals of wildtype or *Tspo* KO group were used for the measurement.

### 2.5. Immunohistochemistry

Mouse eye cryosections were air-dried for 10 min and rehydrated by washing buffer (0.025% Triton X-100 in 1×TBS) two times (5 min each time), then blocked with blocking buffer (0.3% Triton X-100/5% sheep serum in 1×TBS) for 1 h at room temperature. The sections were incubated with anti-Iba-1 primary antibody in blocking buffer overnight at 4 °C, washed 3 times by washing buffer (5 min each), followed by incubation with AF488 secondary antibody (Thermo Fisher Scientific, Paisley, UK) for 1 h at room temperature. Slides were counterstained with 4′,6-diamidino-2-phenylindole (DAPI) (Thermo Fisher Scientific, Paisley, UK) after being washed 5 times (5 min each) using washing buffer. Images were taken under confocal microscopy. Intensity of microglia in ONL was quantified following our previous description [24].

### 2.6. Measurement of Cholesterol Efflux in Mouse Primary Retinal Pigment Epithelium (RPE) Cells

Mouse primary RPE cells were isolated from WT and *Tspo* homozygous KO mice according to our previous description [18]. Isolated RPE cells were seeded on 12-well plates and labelled with [^3^H]cholesterol for 24 h with 2% bovine serum albumin in serum-free culture media. Efflux was initiated by the addition of serum-free DMEM/F12 containing apolipoproteins A-I (ApoA-I, 10 mg/mL), high-density lipoprotein (HDL, 20 mg/mL) or human serum (1%, *v/v*) and cultured for 24 h. Cholesterol efflux was then calculated as an expression of percentage of cholesterol efflux to each of the acceptors as follows: % efflux = (disintegrations per minute (DPM) media/DPM Media + DPM Cells) × 100.

### 2.7. Gene Expression

Total RNAs were extracted from brain, retina and RPE/choroid/sclera using Tri Reagent (Sigma, Dorset, UK), according to the manufacturer’s protocol. cDNAs were synthesized using a High-Capacity cDNA Reverse Transcription Kit with RNAase inhibitor (Thermo Fisher Scientific), following the manufacturer’s guidance. Quantitative real-time polymerase chain reaction (qRT-PCR) was performed using a Platinum Syber Green QPCR Super Mix-UDG w/ROX kit (Thermo Fisher Scientific, Inchinnan, Scotland). Briefly, the reaction was carried out in triplicate in a 10 µL reaction volume containing 5 µL of Syber Green mix, 2 µL of cDNA sample (~100 ng per 10 µL reaction), 0.2 µL of 10 µM forward and reverse primers (Table S1) and 2.6 µL of nuclease-free water to quantify gene expression under the following temperature conditions: 50 °C for 2 min (UDG incubation), followed by the enzyme activation step at 95 °C for 2 min and the amplification step of 40 cycles including DNA denaturation at 95 °C for 15 s and primer annealing at 60 °C for 15 s. The quantification was performed on a Bio-Rad CFX96 Real-Time PCR Detection System (Bio-Rad, Watford, UK). The fluorescence signals were detected at the end of the 60 °C step and the assay validity was examined on the basis of the melting curve between 60 and 95 °C for 1 s per 1 °C, followed by each run. The relative expression of candidate genes was calculated based on the formula: 2^−ΔΔCT^, which was normalized by the housekeeping gene.

### 2.8. Quantification of Total Cholesterol, Phospholipid and Triglyceride in Mouse Tissues

Mouse tissues (brain, retina and RPE/choroid/sclera) were homogenized with a lipid extraction buffer: Hexane: isopropanol (3:2) and incubated for 30 min at room temperature. The organic solvents were centrifuged at 1500× *g* for 10 min and the supernatant was collected and dried at 50 °C. The dried solvent was dissolved with 1× reaction buffer. Total cellular cholesterol and phospholipid were measured using an Amplex^®^ Red Cholesterol Assay Kit (Alfa Aesar, Heysham, UK) and a Phospholipid Assay Kit (Sigma, Dorset, UK), respectively, according to the manufacturer’s guidance. For measurement of triglyceride, mouse tissues were homogenized in buffer containing 5 volumes of isopropanol, 2 volumes of water and 2 volumes of Triton X-100 and centrifuged for 4 min at 13,000 rpm. The supernatants were collected and subjected to triglyceride measurement using an EnzyChrom Triglyceride assay kit (BioAssay system, Hayward, CA, USA), according to the manufacturer’s protocol.

### 2.9. Enzyme-Linked Immunosorbent Assay (ELISA)

ELISA was performed to determine the concentration of IL-1β, IL-6 and TNFa in wildtype and *Tspo* KO mouse tissues. The IL-1 β, IL-6 and TNFa ELISA kits were purchased from PeproTech, UK. The ELISA assay for IL-1β, IL-6 and TNFa was performed in a Nunc-Immuno™ MicroWell™ 96-well solid plate (Sigma, Dorset, UK), according to the manufacturer’s protocol. In brief, the plate was coated with the captured antibody (100 µL/well, 3 µg/mL) and incubated overnight at room temperature. The uncaptured antibody was aspirated and the plate was washed 4 times with the washing buffer. The plate was incubated with the blocking buffer (300 µL/well) for 2 h at room temperature, then washed 4 times with the washing buffer. Further, 100 µL/well of working standard solution of IL-1β, IL-6 and TNFa (0, 10, 100, 1000 pg/mL) and 100 µL/well of unknown samples (tissue extraction) were added, and the plate was incubated at room temperature for 2 h. All liquid was removed and the plate was washed 4 times with the washing buffer. The plate was incubated with respective antibodies, incubated at room temperature for 2 h, then washed 4 times with the washing buffer. The plate was incubated with Avidin-HRP conjugate (100 µL/well, 1.25 µg/mL) in darkness at room temperature for 45 min. The unbound Avidin-HRP conjugate was aspirated and the plate was washed 4 times with the washing buffer. Finally, the plate was incubated with 100 µL/well of ABTS Liquid Substrate (Sigma, Dorset, UK), and the color change was monitored every 5 min for a period of 30 min. The absorbance (OD) was measured at 405 nm with wavelength correction set at 650 nm. The standard curve was constructed on the basis of the mean absorbance for each standard concentration (X *axis*) against the target standard concentration (Y *axis*). The concentration of unknown samples (pg/mL) was calculated based on the straight-line equation obtained from the linear-regression trendline according to Y = mX + c (where Y = OD of unknown sample, m = the slope value, X = the concentration of unknown sample and c = intercept). All experiments were repeated 3 times in triplicate.

### 2.10. Statistical Analysis

Data were analyzed by statistical significance using analysis of variance (ANOVA) and *t*-test; after that, appropriate post hoc tests (Bonferroni) using Prism software (version 6.0 from GraphPad Software Inc., San Diego, CA, USA) were conducted. All data were from a minimum of three independent experiments in triplicate unless otherwise stated. The data are presented as mean ± SD. *p* < 0.05 means significant. * *p* < 0.05, ** *p* < 0.01, *** *p* < 0.001, **** *p* < 0.0001.

## 3. Results

### 3.1. Confirmation of TSPO Deletion

Previously, *Tspo* KO mice were generated by deletion of exons 2 and 3 of *Tspo* gene [22]. To genotype *Tspo* KO mice, primers flanking exons 2 and 3 were used. We amplified a 2697 bp fragment in WT and a fragment of 872 bp fragment in KO mice as predicted (Figure S1A). Further, we examined TSPO protein in mouse tissues by Western blot. We found that WT mouse RPE/choroid/sclera expressed high levels of TSPO protein, with lower levels found in the neural retinas (Figures S1B and S2A), consistent with our previous report [18]. However, we did not detect TSPO protein in WT mouse brain (Figures S1C and S2B), though Betlazar et al. (2018) reported low levels of TSPO expression in mouse brain detected as by immunohistochemistry [25]. It is possible that low levels of TSPO protein in the whole brain lysates are undetectable by Western blot.

### 3.2. No Morphological Changes in Tspo KO Retinas

**Figure 1:**
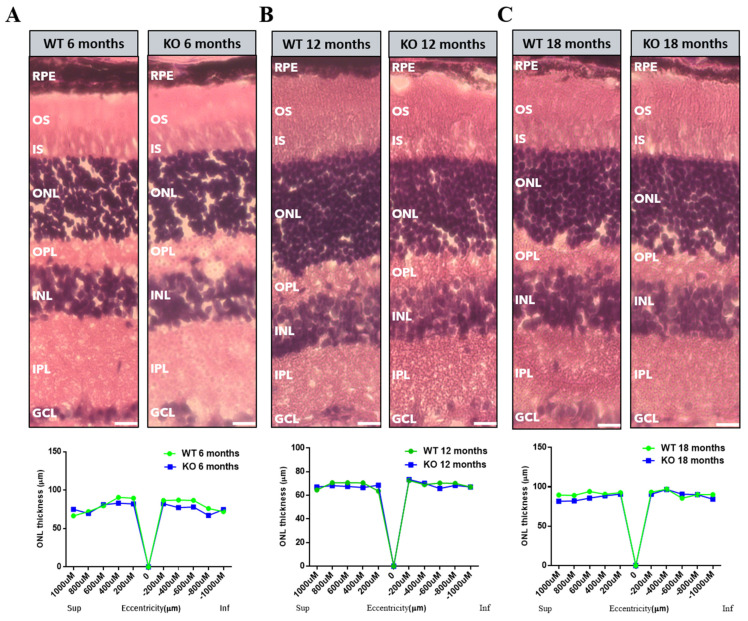
Retinal morphology in WT and *Tspo* KO mice. Histological examination with haematoxylin and eosin staining showing normal retinal morphology and no significant difference in thickness of outer nuclear layer (ONL) in WT and *Tspo* KO mice at 6 (**A**), 12 (**B**) and 18 mon (**C**) of age. Graphs show the thickness of ONL on both the superior (Sup) and inferior (Inf) sides of the retina (*n* = 5). INL: inner nuclear layer; IPL: inner plexiform layer; ONH: optic nerve head; ONL: outer nuclear layer; OPL: outer plexiform layer; RPE: retinal pigment epithelial cells. Statistical comparisons were performed by a non-parametric *t*-test following by Wilcoxon matched-pairs signed rank test.

To assess whether TSPO deletion affects retinal structure, we performed Haematoxylin and Eosin staining on cryosections of eyes from *Tspo* KO and WT mice at 6, 12 and 18 months old (Figure 1). No gross morphological differences between WT and *Tspo* KO mouse retinas were observed by light microscopy. To investigate if there was any photoreceptor loss, we measured the thickness of the outer nuclear layer at five different points along the superior and inferior regions of the retinas in WT and *Tspo* KO mice. No significant difference in the thickness of outer nuclear layer between WT and *Tspo* KO mice was observed at all age points (Figure 1).

### 3.3. Cholesterol Efflux Reduced in Tspo KO Mouse RPE Cells

**Figure 2:**
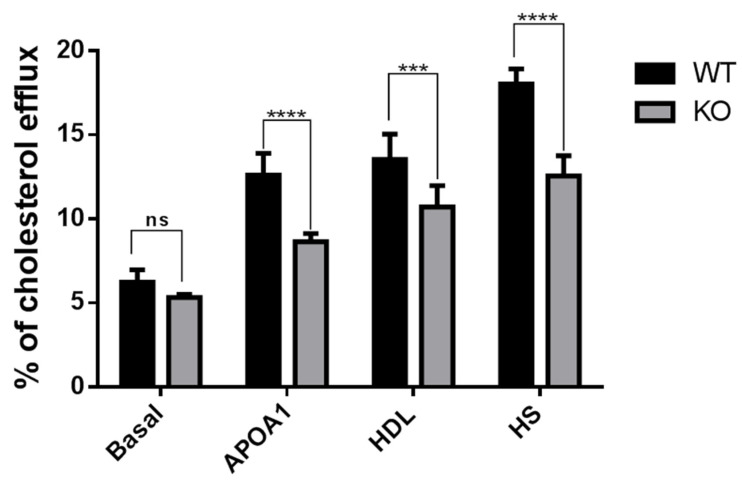
Decreased cholesterol efflux in Tspo KO mouse RPE cells. Primary RPE cell derived from wildtype (WT) and *Tspo* KO mice at 6 months old were labelled with 0.5 lCi/mL [^3^H]cholesterol for 24 h followed by 24 h incubation with or without apolipoproteins A–I (ApoA-I, 10 μg/mL), HDL (20 μg/mL) and human serum (HS, 1% *v*/*v*). After incubation, the percentage of [^3^H]cholesterol efflux was measured. Data were collected from three independent experiments and analyzed by two-way ANOVA followed by Bonferroni test: NS: no significance; *** *p* < 0.001, **** *p* < 0.0001.

Our previous study demonstrated that loss of TSPO in human RPE cells resulted in cholesterol efflux defects [18]. Next, we examined the effect of TSPO deletion on cholesterol efflux in mouse primary RPE cells. We observed that the percentage of [^3^H]cholesterol efflux to apoA-I, HDL, or human serum was significantly decreased in *Tspo* KO mouse RPE cells when compared to that of WT RPE cells (Figure 2).

### 3.4. Increased Lipid Accumulation in Tspo KO Mouse Tissues

**Figure 3:**
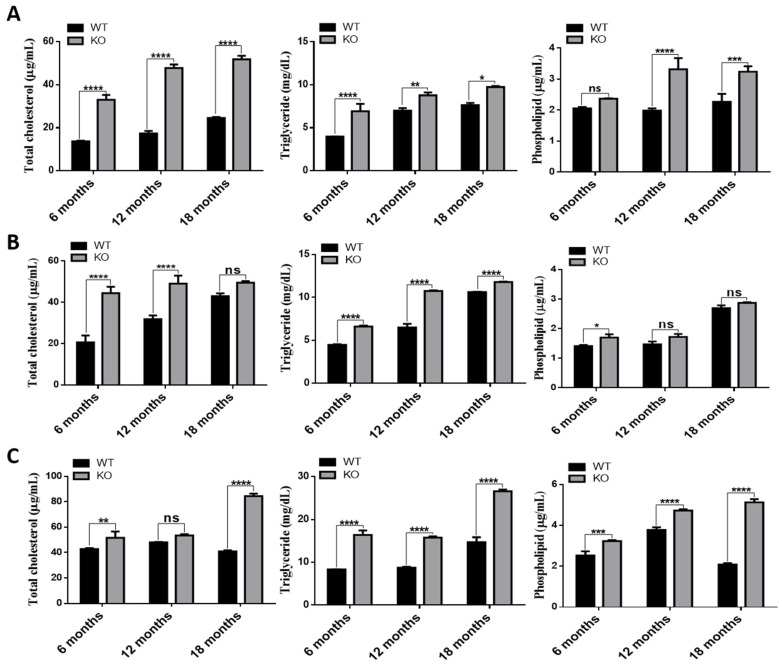
Total cholesterol, Triglycerides and phospholipids in RPE/choroid/sclera (**A**), retina (**B**) and brain (**C**) brain of wildtype and *Tspo* KO mice (6, 12 and 18 months old) were measured and normalized to total protein contents. Data were collected from three independent experiments and analyzed by two-way ANOVA followed by Bonferroni test. NS: no significance; * *p* < 0.05, ** *p* < 0.01, *** *p* < 0.001, **** *p* < 0.0001.

Previously, we reported that loss of TSPO caused accumulation of intracellular oxidized LDL in human RPE cells [18]. Here, we measured cholesterol mass, triglycerides and phospholipids in RPE/choroid/sclera, retina and brain of WT and *Tspo* KO mice at the ages of 6, 12 and 18 months. The contents of cholesterol, triglycerides and phospholipids in RPE/choroid/sclera of *Tspo* KO mice were significantly increased compared to that of WT mice (Figure 3A). In *Tspo* KO retinas, cholesterol levels were significantly higher at 6 and 12 months old but not at 18 months old when compared to that of WT mice. Triglyceride levels in *Tspo* KO retinas were notably increased at all age points when compared to that of WT animals. Phospholipids in *Tspo* KO retinas were only significantly increased at the age of 6 months when compared to WT animals (Figure 3B). Similar observations were seen in *Tspo* KO brains. Cholesterol levels in *Tspo* KO brains were significantly increased at 6 and 18 months old but not at 12 months old, compared to that of WT animals. Both triglycerides and phospholipids were significantly higher in *Tspo* KO brains at all ages when compared to those of WT mice (Figure 3C).

### 3.5. Deletion of TSPO Decreased Expression of Cholesterol Homeostasis Genes

**Figure 4:**
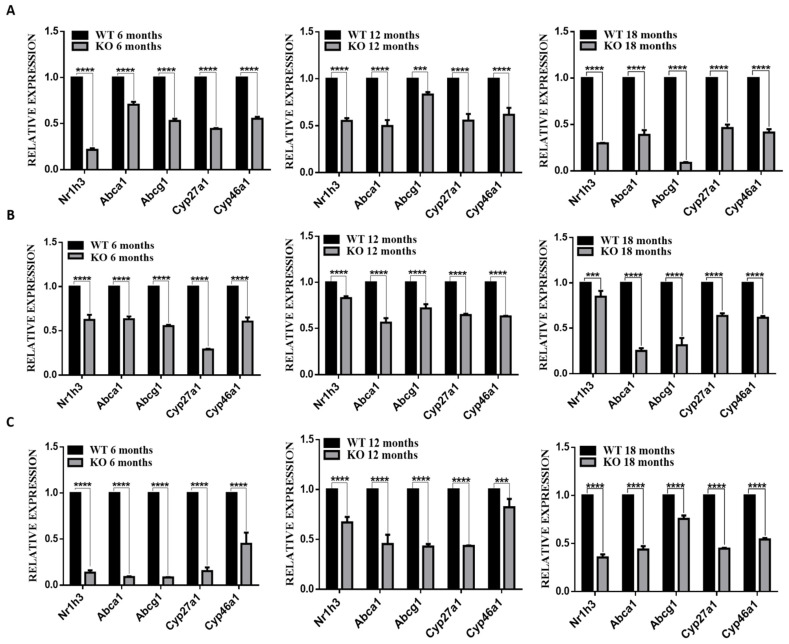
The expression of cholesterol homeostasis genes: *Nr1h3*, *Abca1*, *Abcg1*, *Cyp27a1* and *Cyp46a1* in RPE/choroid/sclera (**A**), retina (**B**) and brain (**C**) of wildtype (WT) and *Tspo* KO mice at 6, 12 and 18 months old. The qRT-PCR data were collected as ct (Cycle threshold) values and normalised to *Gapdh* then analyzed by 2^−ΔΔCT^ formula. The fold changes of each gene were presented as means ± SD and analyzed by two-way ANOVA with the appropriate post hoc test Bonferroni multiple comparisons tests (*n* = 5). *** *p* < 0.001, **** *p* < 0.0001.

Since deletion of TSPO caused cholesterol efflux defects in RPE cells and increased cholesterol levels in RPE/choroid/sclera, retinas and brains (Figure 2 and Figure 3), we examined expression of genes associated with cholesterol metabolism and trafficking. The expression of *Nr1h3* (encoding liver X receptor alpha, LXRa), *Abca1*, *Abcg1*, *Cyp27a1* and *Cyp46a1* was significantly downregulated in the RPE/choroid/sclera and retina of *Tspo* KO mice at 6, 12 and 18 months old compared to WT mice (Figure 3A,B). Similarly, expression of these genes was markedly decreased in brains of *Tspo* KO mice at the ages of 6, 12 and 18 months compared to that of WT mice (Figure 4C).

### 3.6. Deletion of TSPO Increased Inflammation in The Tissues of Tspo KO Mice

**Figure 5:**
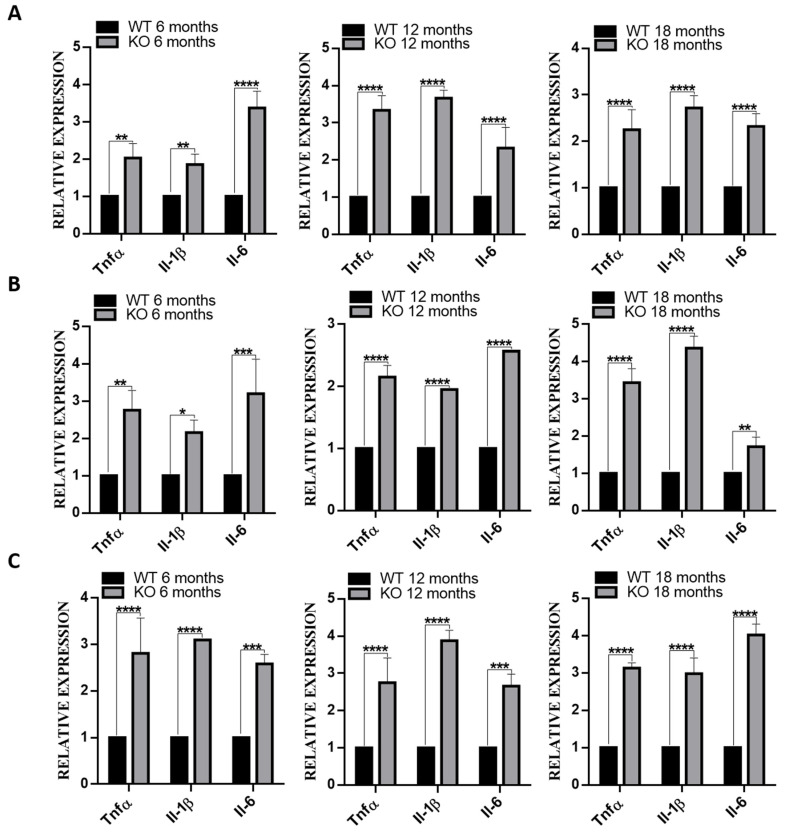
The expression of inflammation genes, *Tnfa*, *Il-1β* and *Il-6*, in RPE/choroid/sclera (**A**), retina (**B**) and brain (**C**) of WT and *Tspo* KO mice at the ages of 6, 12 and 18 months. The qRT-PCR data were collected as ct (cycle threshold) values and normalised to *Gapdh*, then analyzed by 2^−ΔΔCT^ formula. The fold changes of each gene were presented as means ± SD and analyzed by two-way ANOVA with the appropriate post hoc test Bonferroni multiple comparisons tests (*n* = 5). * *p* < 0.05, ** *p* < 0.01, *** *p* < 0.001, **** *p* < 0.0001.

**Figure 6:**
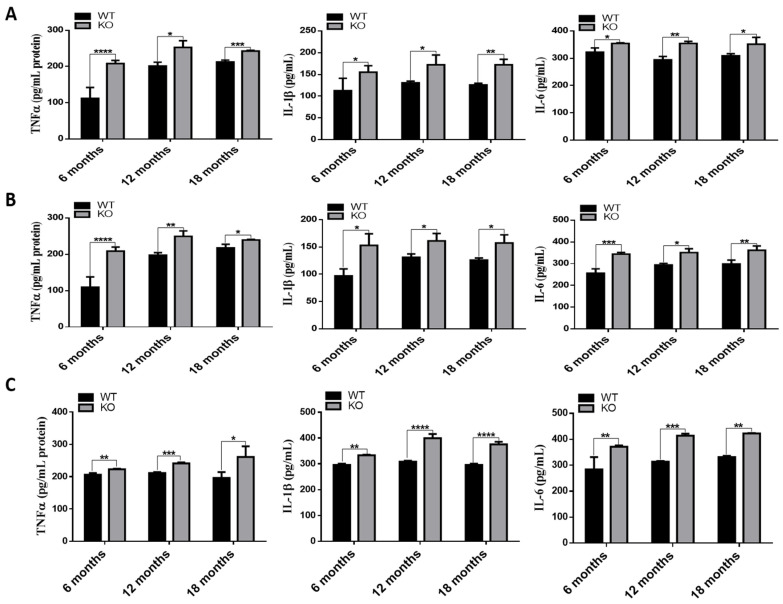
The levels of the inflammatory cytokines: TNFα, IL-1β and IL-6, in RPE/choroid/sclera (**A**), retina (**B**) and brain (**C**) of WT and *Tspo* KO mice at the ages of 6, 12 and 18 months. The data were presented as means ± SD and analyzed by two-way ANOVA with the appropriate post hoc test Bonferroni multiple comparisons tests (*n* = 5). * *p* < 0.05, ** *p* < 0.01, *** *p* < 0.001, **** *p* < 0.0001.

Since increased inflammation was observed in human RPE cells with TSPO deletion [18], we examined the expression of inflammatory genes, *Tnfa*, *Il-1β* and *Il-6*, in RPE/choroid/sclera, retina and brain of WT and *Tspo* KO mice at 6, 12 and 18 months old. The mRNA levels of these genes were markedly higher in RPE/choroid/sclera, retina and brain of *Tspo* KO mice compared to that of WT mice (Figure 5). We also measured TNFα, IL-1β and IL-6 protein by ELISA and found that levels of these inflammatory cytokines were significantly increased in RPE/choroid/sclera, retina and brain of *Tspo* KO mice at 6, 12 and 18 months old compared to that of WT mice (Figure 6)

### 3.7. Microglial Activation in Tspo KO Mouse Retina

**Figure 7:**
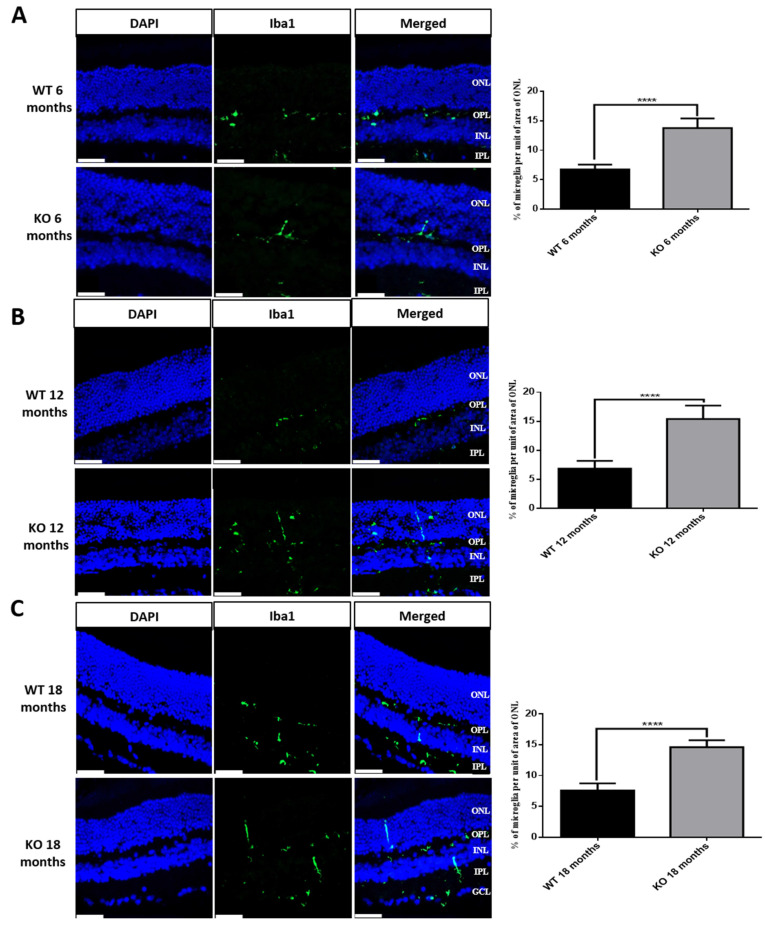
Microglia in the retinas of wiltype and *Tspo* KO mice at the ages of 6 (**A**), 12 (**B**) and 18 (**C**) months. Immunostaining of microglia marker: Iba-1 (green), was performed with cryosections from wildtype and *Tspo* KO mouse eyes. The intensity of microglia in the outer nuclear layer was quantified. Data were analyzed by *t*-test followed by Bonferroni test (*n* = 5). INL: inner nuclear layer; IPL: inner plexiform layer; ONH: optic nerve head; ONL: outer nuclear layer; OPL: outer plexiform layer; RPE: retinal pigment epithelial cells. **** *p* < 0.0001. Scale bar, 10 µm.

Microglia are resident macrophages in the central nervous system, including the retina, where microglia can be activated under stress/pathological conditions [26]. TSPO is thought to mediate neuroinflammation, including microglial activation [16]. To examine whether there is microglial activation in *Tspo* KO mouse retinas, a biomarker (Iba-1) for microglia was detected by immunohistochemistry in cryosections from WT and *Tspo* KO mouse eyes. We observed that microglia were activated and migrated into the outer nuclear layer of *Tspo* KO retinas at the ages of 6, 12 and 18 months; however, in WT retinas, microglia were restricted to outer and inner plexiform layers (Figure 7). TMEM119 is another microglial biomarker, expressed at a higher level in rd1 mouse retina compared to that of WT mice [27]. We measured *Tmem119* mRNA in the RPE/choroid/sclera and retinas of WT and *Tspo* KO mice at 6, 12 and 18 months old, demonstrating that expression of *Tmem119* in *Tspo* KO RPE/choroid/sclera and retinas was significantly increased when compared to that of WT mice (Figure S3).

## 4. Discussion

As a multiple-function protein, TSPO was initially thought to play a critical role in steroidogenesis, via transporting cholesterol from the mitochondrial outer membrane to the inner membrane, where cholesterol is cleaved by CYP11A1 to produce pregneolone, the precursor of steroids. Early work demonstrated that TSPO ligands promote steroid generation and knockdown or disruption of TSPO decreases production of steroid hormone [28,29,30,31]. However, some recent work related to TSPO-associated steroidogenesis is controversial. Data from Dr. Selvaraj’s lab demonstrated that global or conditional knockout of *Tspo* in mice had no effect on steroid production [22,24]. Data from Drs. Papadopoulos and Higuchi’s laboratories showed that global or conditional deletion of TSPO in mice resulted in steroidogenic abnormalities [32,33,34]. Owen et al. reported that knockout of *Tspo* in rats also caused impaired steroid synthesis, and humans carrying the rs6971 polymorphism (Ala147Thr) had decreased adrenocorticotropic hormone-induced corticosteroid levels [35]. The inconsistent results from individual studies are possibly due to different genetic backgrounds or approaches to create the KO lines.

We were the first to investigate TSPO function in the retinas, demonstrating that TSPO is expressed at high levels in human and mouse RPE cells, and its expression is markedly decreased in aged-mouse RPE. We also found that TSPO mediated mitochondrial cholesterol efflux in RPE cells and that deletion of TSPO resulted in cholesterol efflux defects and accumulation of cholesterol [18]. Here, *Tspo* knockout mouse RPE cells also showed impaired cholesterol efflux (Figure 2). There were also significantly higher levels of cholesterol, triglycerides and phospholipids and downregulation of cholesterol homeostasis genes in *Tspo* KO RPE/choroid/sclera, retina and brain (Figure 3 and Figure 4), suggesting that TSPO mediates cholesterol metabolism and transport in the central nervous system.

TSPO deletion in human RPE cells caused increased uptake and accumulation of oxidized LDL, which induced inflammation [18]. Accumulated cholesterol can be oxidized to form oxysterols, some of which, such as 7-ketocholesterol, are toxic and induce inflammation and angiogenesis. 7-ketocholesterol is commonly presented in oxidized LDL, both 7-ketocholesterol and oxidized LDL are enriched in drusen, a key clinical feature of AMD [36]. TSPO ligands also suppressed production of inflammatory cytokines (IL-1β, IL-6, TNFα and VEGF) induced by oxidized LDL in choroidal endothelial cells [19]. Actually, TSPO ligands have demonstrated protection in various neurodegenerative disorders by counteracting inflammation [37,38]. *Tspo* KO mouse RPE/choroid/sclera, retina and brain had higher expression (mRNA and protein) of inflammatory cytokines: IL-1β, IL-6 and TNFα (Figure 5 and Figure 6). Elevated expression of these cytokines may be a direct result of cholesterol accumulation and its derived oxysterols, which warrants further investigation.

Microglial activation plays an important role in the pathogenesis and progression of neurodegenerative diseases, including retinal degeneration [26]. TSPO stands out as a possible biomarker in the arena of neuroinflammation, with ligands under scrutiny at present in the arena of clinical neuroimaging [14]. However, the mechanism which impels the upregulation of TSPO during the disorders remains ambiguous, as does the role of TSPO in the activation of microglia. TSPO ligand, XBD173, has been shown to inhibit microglial activation in a mouse microglial cell line and in living-mouse retinal explants, suppressing inflammation [39]. XBD173 also inhibited microglial activation and prevented retinal degeneration in mice exposed to acute light damage [40]. We also found microglial activation in *Tspo* KO mouse retinas at the ages of 6, 12 and 18 months (Figure 7); the underlying mechanism for the activation needs further attention.

As a complex disease, the development of AMD pathology is poorly understood. It is believed that cholesterol homeostasis, extracellular matrix remodelling, complement activation and angiogenesis are involved in the pathogenesis and progression of AMD [4]. Although *Tspo* KO mice demonstrated some AMD pathological features such as cholesterol efflux defect, accumulation of cholesterol in the retina, increased inflammation and microglial activation, the thickness of outer nuclear layer was not different between WT and *Tspo* KO mice even at the age of 18 months (Figure 1), suggesting no notable photoreceptor cell death in *Tspo* KO retinas. Further examination is required to explore whether there are any subtle pathological changes such as sub-RPE deposits and RPE atrophy in *Tspo* KO mouse retinas using transmission electron microscopy. The other possibility is that the retinal pathology in *Tspo* KO mice may develop much later. The complement factor h gene (*CFH*) is the major gene associated with AMD (Hageman et al., 2005; Haines et al., 2005; Klein et al., 2005) [41,42,43]. *Cfh* genetic altered mouse models are widely used to understand the pathological mechanisms of AMD. *Cfh^−/−^* mice only displays subtle retinal pathology such as increased subretinal autofluorescence and disorganized photoreceptor outer segments at the age of 24 months old [44]. However, *Cfh^−/−^* mice develop more dominant retinal phenotypes when fed with a high-fat diet [12,45]. So, it is necessary to further examine retinal pathology in *Tspo* KO mice fed with a high-fat diet.

## 5. Conclusions

The current study demonstrates that deletion of TSPO in mice resulted in cholesterol efflux defect in RPE cells, increased lipids (cholesterol, triglyceride and phospholipid) in the retinas, decreased expression of cholesterol-associated genes, increased inflammation, and microglial activation. We believe that further studies of *Tspo* KO mouse model will help to understand TSPO-associated function in the retina and to develop new treatment for AMD.

## Data Availability

All data generated from this study are included in this manuscript.

## Supplementary Materials

The following are available online at https://www.mdpi.com/article/10.3390/cells10113066/s1, Figure S1: Genotyping of wildtype (flx) and TSPO KO (DD4X) mouse by PCR, Figure S2: the original gel images, Figure S3: the expression of microglial biomarker, *Tmem119* gene, Table S1: Primers for qRT-PCR.
