# Supplementary material for: Deletion of TSPO Causes Dysregulation of Cholesterol Metabolism in Mouse Retina
Source: Cells. 2021 Nov 7;10(11):3066. doi: 10.3390/cells10113066 (PMC8621976; doi:10.3390/cells10113066)
Supplement: Supplementary file 1 [file cells-10-03066-s001.zip › cells-1456954-supplementary.pdf]

cells

MDPI

## Supplementary data

**Table S1.** Primers for qRT-PCR.

| Gene | Forward Primer (5′-3′) | Forward Primer (5′-3′) |
|---|---|---|
| *Il-1 β* | CAGGCAGGCAGTATCACTCA | AGCTCATATGGGTCCGACAG |
| *Tnf-α* | ACGTGGAACTGGCAGAAGAG | AGGGTCTGGGCCATAGAACT |
| *Il-6* | TACCACTTCACAAGTCG | CTGCAAGTGCATCATCG |
| *Nr1h3* (*coding* LXRα) | CCCTAGCCTTTCCCCAAATTGC | CGGAAGAATCCCTTGCAACC |
| *Abca1* | AGTTTCGGTATGGCGGGTTT | AGCATGCCAGCCCTTGTTAT |
| *Abcg1* | ACCTACCACAACCCAGCAGACTTT | GGTGCCAAAGAAACGGGTTCACAT |
| *Cyp27a1* | GCCTTGCACAAGGAAGTGACT | CGCAGGGTCTCCTTAATCACA |
| *Cyp46a1* | CCCTAGCCTTTCCCCAAATTGC | CGGAAGAATCCCTTGCAACC |
| *Tmem119* | *CCTTCACCCAGAGCTGGTTC* | *GGCTACATCCTCCAGGAAGG* |
| *Gapdh* | GTCTCCTGCGACTTCAGC | TCATTGTCATACCAGGAAATGAGC |

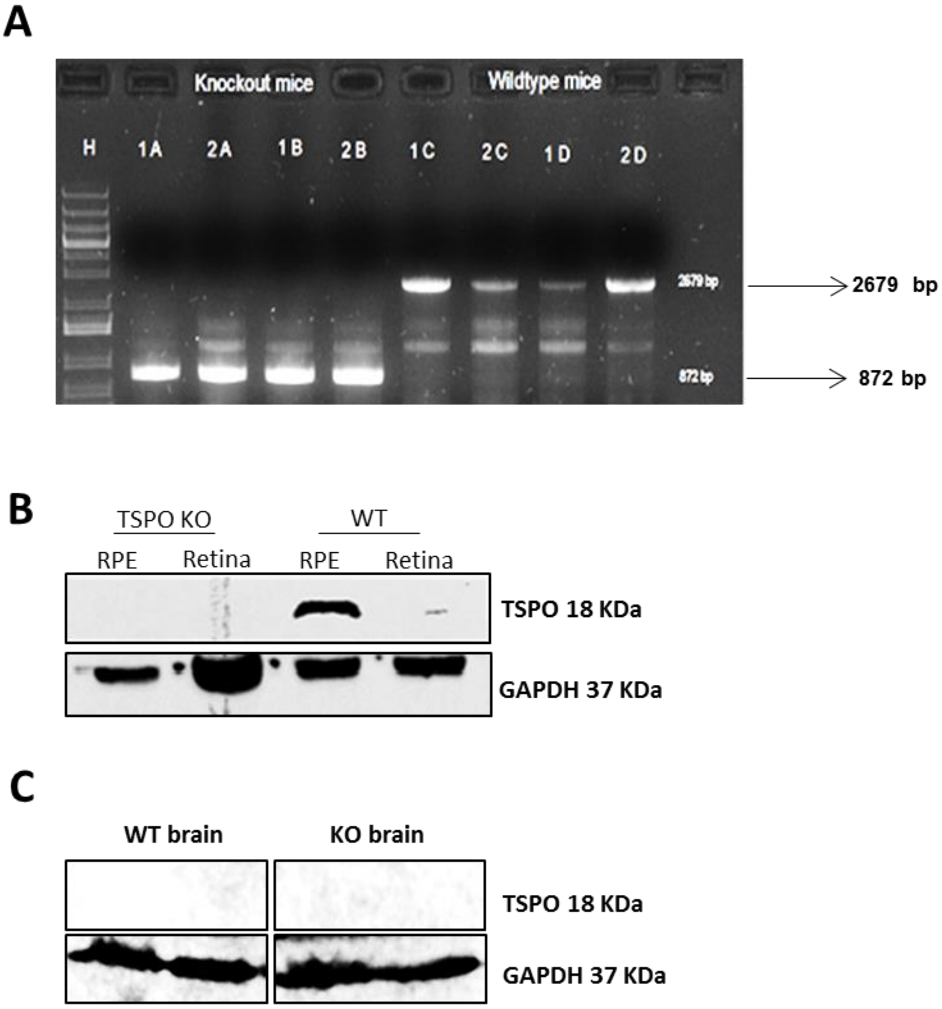


**Figure S1.** Genotyping of wildtype (flx) and TSPO KO (DD4X) mouse by PCR **(A).** Lane 1: 1kb plus DNA ladder; Lane 2-5: KO amplicon gave 872bp band; Lane 6-9: WT amplicon gave 2679bp band. WT and TSPO-KO mice **(B)** RPE, Retina and **(C)** Brain were screened for TSPO protein expression using a monoclonal TSPO antibody. The TSPO-deleted colonies in (RPE, Retina and Brain) were identified by Western blotting.

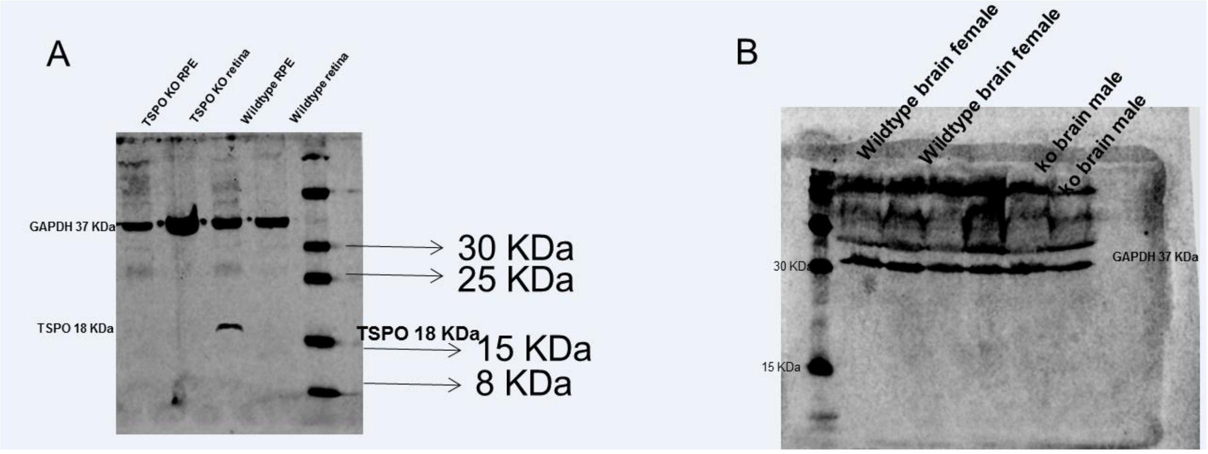


**Figure S2.** The original gel images for data presented in Figure S1B and C.

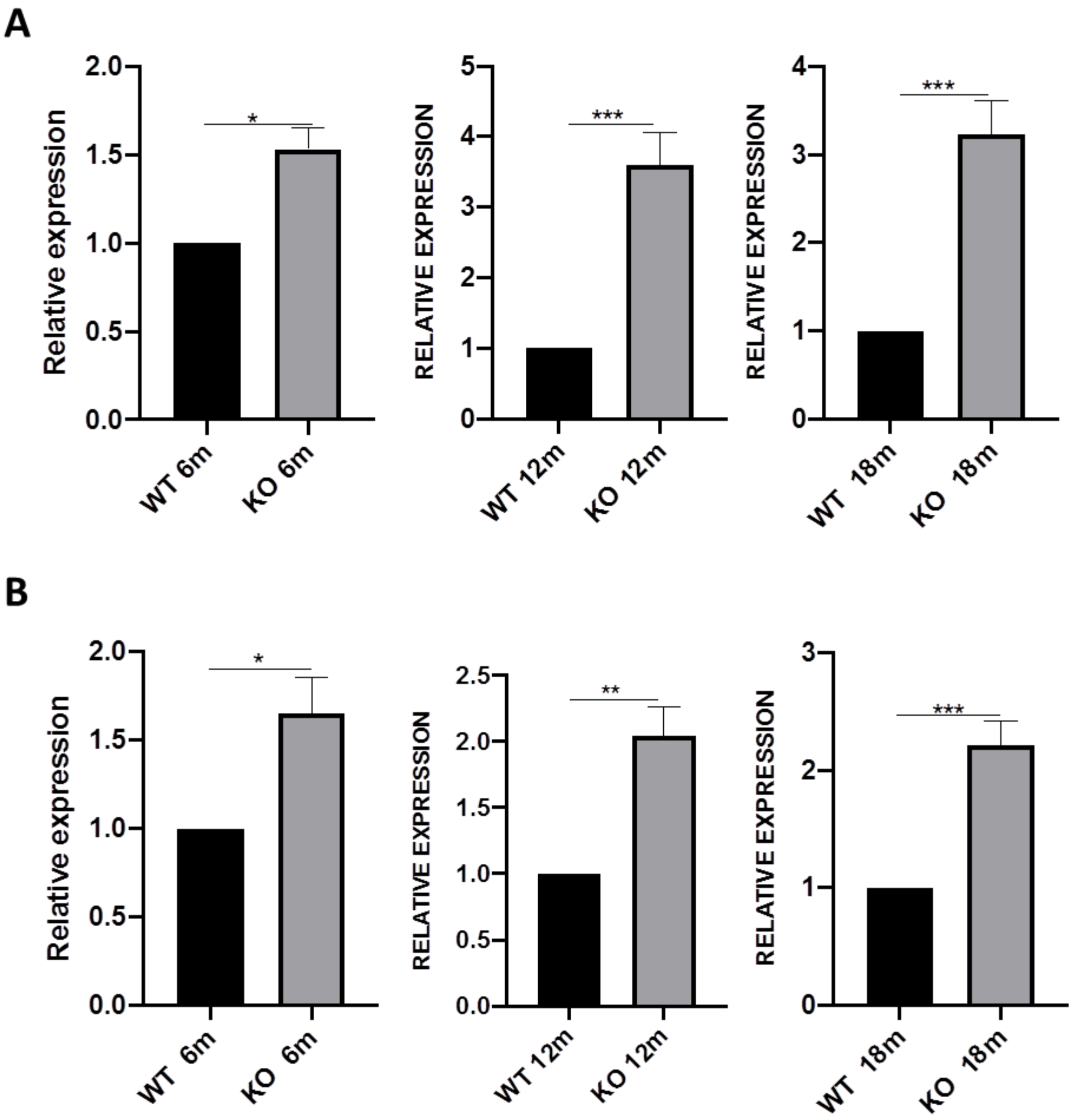


**Figure S3.** The expression of microglial biomarker, *Tmem119* gene, in RPE/choroid/sclera **(A)** and retinas **(B)** of wildtype and Tspo KO mice at age of 6, 12 and 18 months. The qRT-PCR data was collected as ct (Cycle threshold) values and normalised to *Gapdh* then analysed by $2^{-\Delta\Delta CT}$ formula. The data were presented as means ± SD and analysed using a t-test with the appropriate post hoc test Bonferroni multiple comparisons tests. *p<0.05, **P<0.01, ***P<0.001.
